# *MALAT1::PLAG1* Fusion in Pleomorphic Adenoma with Mucinous and Squamous Metaplasia: A Diagnostic Pitfall Mimicking Mucoepidermoid Carcinoma (ex Pleomorphic Adenoma)

**DOI:** 10.1007/s12105-026-01977-1

**Published:** 2026-10-26

**Authors:** Liang Lu, Martina Bradová, Elan Hahn, Iona Leong, Laura J. Tafe, Darcy A. Kerr, Ivan J. Stojanov

**Affiliations:** 1 Department of Pathology and Laboratory Medicine, Dartmouth-Hitchcock Medical Center, Lebanon, NH USA; 2 Department of Pathology, Faculty of Medicine in Pilsen and Faculty Hospital, Charles University, Pilsen, Czech Republic; 3 Bioptic Laboratory, Ltd., Pilsen, Czech Republic; 4 Department of Pathology and Laboratory Medicine, Mount Sinai Hospital, Toronto, ON Canada; 5 Department of Laboratory Medicine and Pathobiology, University of Toronto, Toronto, ON Canada; 6 Faculty of Dentistry, University of Toronto, Toronto, ON USA; 7 Robert J. Tomsich Department of Pathology and Laboratory Medicine, Cleveland Clinic, 9500 Euclid Avenue, Cleveland, OH L15 USA

**Keywords:** Pleomorphic adenoma, Carcinoma ex pleomorphic adenoma, Mucoepidermoid carcinoma, Salivary tumor, *PLAG1*, Genotype–phenotype correlation, Metaplasia

## Abstract

**Purpose:**

Pleomorphic adenoma (PA), the most common salivary neoplasm, is capable of tremendous histologic diversity. Co-occurrence of squamous and mucinous differentiation in PA is a rare phenomenon generally considered within its histologic spectrum, though previously advocated by some as evidence of mucoepidermoid carcinoma (MEC) ex PA. Herein, we describe a multi-institutional series of MEC-like PA with squamous differentiation, mucous cells, cystic change, and *MALAT1::PLAG1* fusion.

**Materials and Methods:**

Retrospective multi-institutional review identified 5 PAs with *MALAT1::PLAG1* fusion. Histopathologic and immunohistochemical slides were reviewed, as well as demographic, clinical, and molecular findings.

**Results:**

Five *MALAT1::PLAG1*-rearranged PA occurred in the parotid gland of 3 female and 2 male patients (median age: 53 yrs, range: 47–58 yrs). Median tumor dimension was 2.5 cm (range: 1.4–3.4 cm). All tumors demonstrated characteristic histologic features of PA as well as areas of cystic squamous and mucinous differentiation resembling MEC (comprising 25–80% of the tumors). There was no capsular invasion or high-grade features. No canonical MEC driver alterations were identified by NGS. No recurrences were identified during available follow-up in 2 patients.

**Conclusion:**

Cystic squamous and mucinous differentiation may occur in PA, representing a potential diagnostic pitfall both in small biopsy specimens, where it can be misdiagnosed as MEC, as well as in resection specimens where it should not be interpreted as MEC ex PA. Instead, apparent association of these histologic features with *MALAT1::PLAG1* fusion suggests a novel genotype–phenotype correlation in PA. Adjunct molecular testing may be valuable in diagnostically challenging cases.

## Introduction

Pleomorphic adenoma (PA) is well-known for its histologic diversity, with tumors capable of demonstrating substantial variability in cellularity, stromal composition, and relative composition of ductal and myoepithelial cell populations [1, 2]. Tumor stroma may contain chondroid, osseous, or lipomatous elements and tumor cells, themselves, may demonstrate squamous or mucinous differentiation [3, 4]. Cystic change of the epithelial component may also occasionally occur [5]. Co-occurrence of squamous and mucinous metaplasia in PA is rare and may resemble mucoepidermoid carcinoma (MEC). Indeed, there are several examples of tumors with these features having been reported as MEC ex PA in the literature [6–12]. These tumors, diagnosed on histologic grounds alone without molecular investigation, have lacked features such as capsular invasion, high-grade cytology or evidence of metastasis or, in other examples, have inadequately substantiated a pre-existing diagnosis of PA [13]. The presence of squamous and mucinous metaplasia in PA has been recognized by other investigators, in contrast, as a diagnostic pitfall within the histologic spectrum of PA [4, 14–17].

More recently, two case reports have identified a novel *MALAT1::PLAG1* fusion in PA with squamous and mucinous metaplasia, further supporting interpretation of these findings as within the histologic spectrum of PA [18, 19]. These findings corroborate earlier work by Skálová, et al. in which PA with squamous and mucinous metaplasia was found to lack *MAML2* alteration despite histologic mimicry of MEC [20]. In this series, we present an additional five cases of PA with squamous metaplasia, mucinous cells, and *MALAT1::PLAG1* fusion, in which we highlight what may represent an emerging genotype–phenotype correlation in PA, draw attention to this potential diagnostic pitfall in small biopsy specimens, and further argue against the notion of MEC ex PA.

## Materials and Methods

With IRB approval (Cleveland Clinic IRB #21–272), 2 cases of PA with squamous and mucinous metaplasia mimicking MEC were prospectively identified by one of the authors (IJS), both of which were sent for molecular testing and found to harbor *MALAT1::PLAG1* fusion. Retrospective review of the authors’ institutional pathology archives for PA with *MALAT1::PLAG1* fusion identified a further 3 cases of PA with *MALAT1::PLAG1* fusion, resulting in a total of 5 cases that were reviewed.

Medical records were reviewed to identify clinical data, including age, sex, tumor location, tumor size, surgical management, and clinical course. All hematoxylin and eosin slides, immunohistochemical slides, and histochemical slides were reviewed. Original formalin-fixed, paraffin-embedded blocks were cut at 4 μm thickness. Immunohistochemical (IHC) staining was performed using the following antibodies: CK7 (OV-TL12/30 mouse monoclonal), p63 (4A4 mouse monoclonal, Ventana Medical Systems), p40 (BC28, Biocare Medical), Dako (Agilent)), S100 (rabbit polyclonal, Dako (Agilent)), SOX10 (BC34 mouse monoclonal isotype IgG1, Biocare Medical), SMA (1A4 mouse monoclonal; Cell marque/VMS), SMMS-1 (SMMS1 mouse monoclonal isotype IgG1/K, Cell Marque), *PLAG1* (3B7 mouse monoclonal, Millipore-Sigma), HMGA2 (EP398 rabbit monoclonal, Bio SB), CD117 (EP10 rabbit monoclonal, VMS), and Ki-67 (30–9 rabbit monoclonal, Ventana Medical Systems). Histochemical staining for mucin was performed with PAS and Alcian blue (pH 2.5).

Molecular analysis on representative macro-dissected formalin-fixed paraffin-embedded tissue was performed on all cases in the course of routine clinical care. Three cases were evaluated with custom in-house anchored multiplex PCR–based targeted RNA sequencing approaches (evaluating 30–629 genes), one case underwent custom whole transcriptome and whole exome sequencing as described previously [21, 22], and one case underwent targeted DNA (523 genes) and RNA (1385) sequencing using the Illumina TruSight Oncology 500 and TruSight RNA Pan-Cancer Panel assays. The reads from complementary DNA libraries were mapped to the human reference genome hg19 (GRCh37). In the four cases evaluated with targeted RNA sequencing approaches, unidirectional gene-specific primers targeted specific exons in genes in order to identify both known and novel oncogenic fusions in head and neck/salivary tumors.

## Results

The cohort consisted of 5 patients, 3 female and 2 male, with a median age of age 53 years (range: 47–58 years) at diagnosis. All tumors arose in the parotid gland and were treated by parotidectomy or extracapsular dissection. Median tumor size was 2.5 cm (range: 1.4–3.4 cm). Three patients were lost to follow up, and in the remaining 2 patients there were no documented recurrences or metastases upon follow-up periods of 8 and 25 months. Clinical data are summarized in Table 1.

**Table 1:** Clinicopathologic and immunohistochemical findings in *MALAT1::PLAG1*-rearranged pleomorphic adenoma

1	54	F	3.4	80	NP	NP	NP	NP	NP	NP	NP	NP	NP	+ (incl. in MEC-like areas)	NP	+	NP	N/A
2	53	F	2.5	25	+	+	+	+ (incl. focally in MEC-like areas)	+ (incl. focally in MEC-like areas)	+ (focal)	+ (focal)	+ (in ductal cells)	NP	+ (incl. in MEC-like areas)	-	NP	< 3%	N/A
3	47	M	1.4	50	NP	+	+	+ (incl. focally in MEC-like areas)	+ (incl. focally in MEC-like areas)	NP	NP	NP	NP	+	NP	+	5%	25 mos. ANED
4	58	M	1.4	80	NP	NP	NP	NP	NP	NP	NP	NP	NP	NP	NP	NP	NP	N/A
5	50	F	2.5	60	+	NP	+	+ (incl. focally in MEC-like areas)	NP	NP	NP	NP	NP	+	NP	+	NP	8 mos ANED

NP, not performed; N/A, not available; ANED, alive with no evidence of disease

**Fig. 1:**
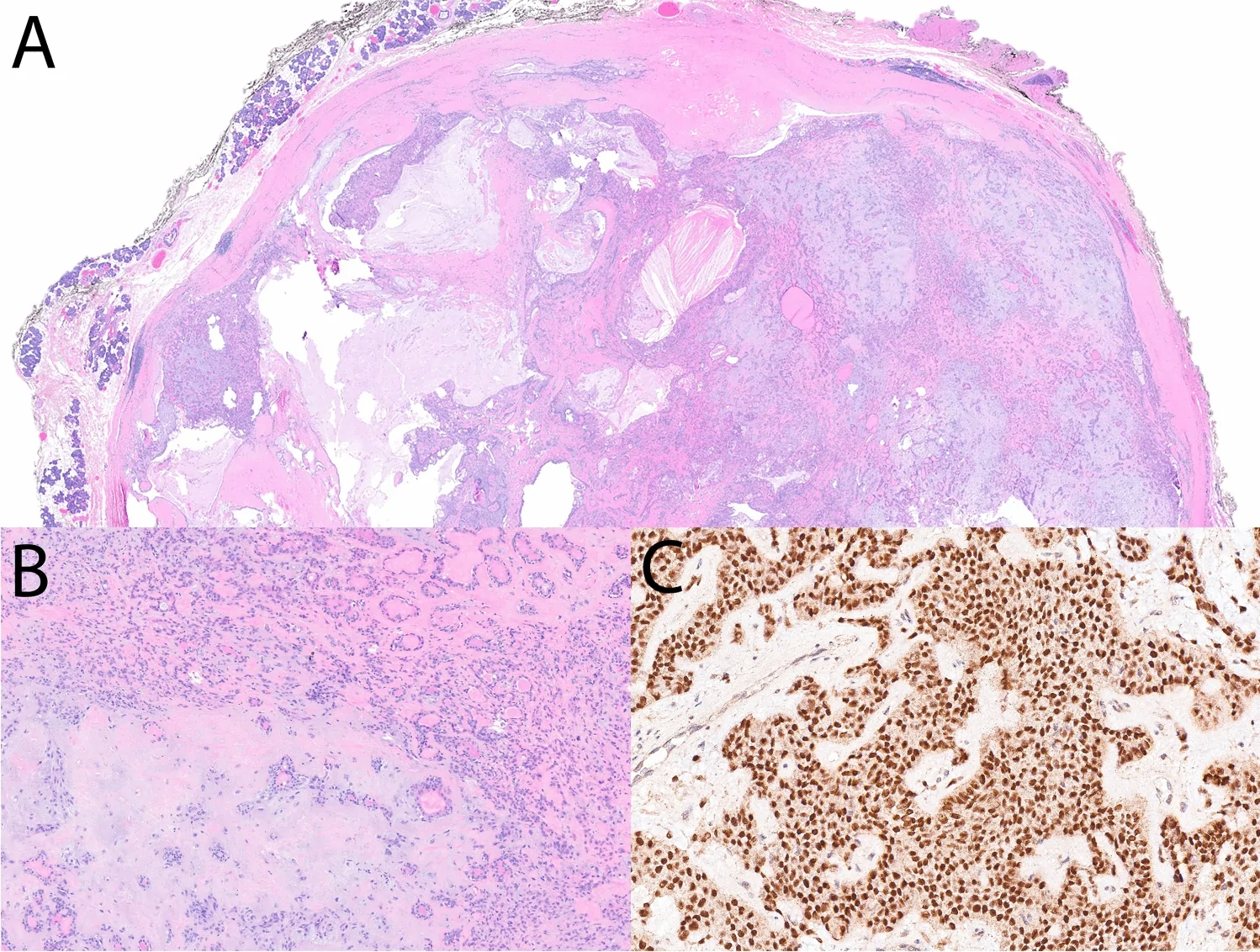
All tumors were circumscribed and/or encapsulated (**A**) with characteristic features of PA, namely, an admixture of ductal and myoepithelial cells within chondromyxoid or fibrous stroma (**B**). *PLAG1* immunohistochemistry, when performed, was strongly positive in myoepithelial cells (**C**)

**Fig. 2:**
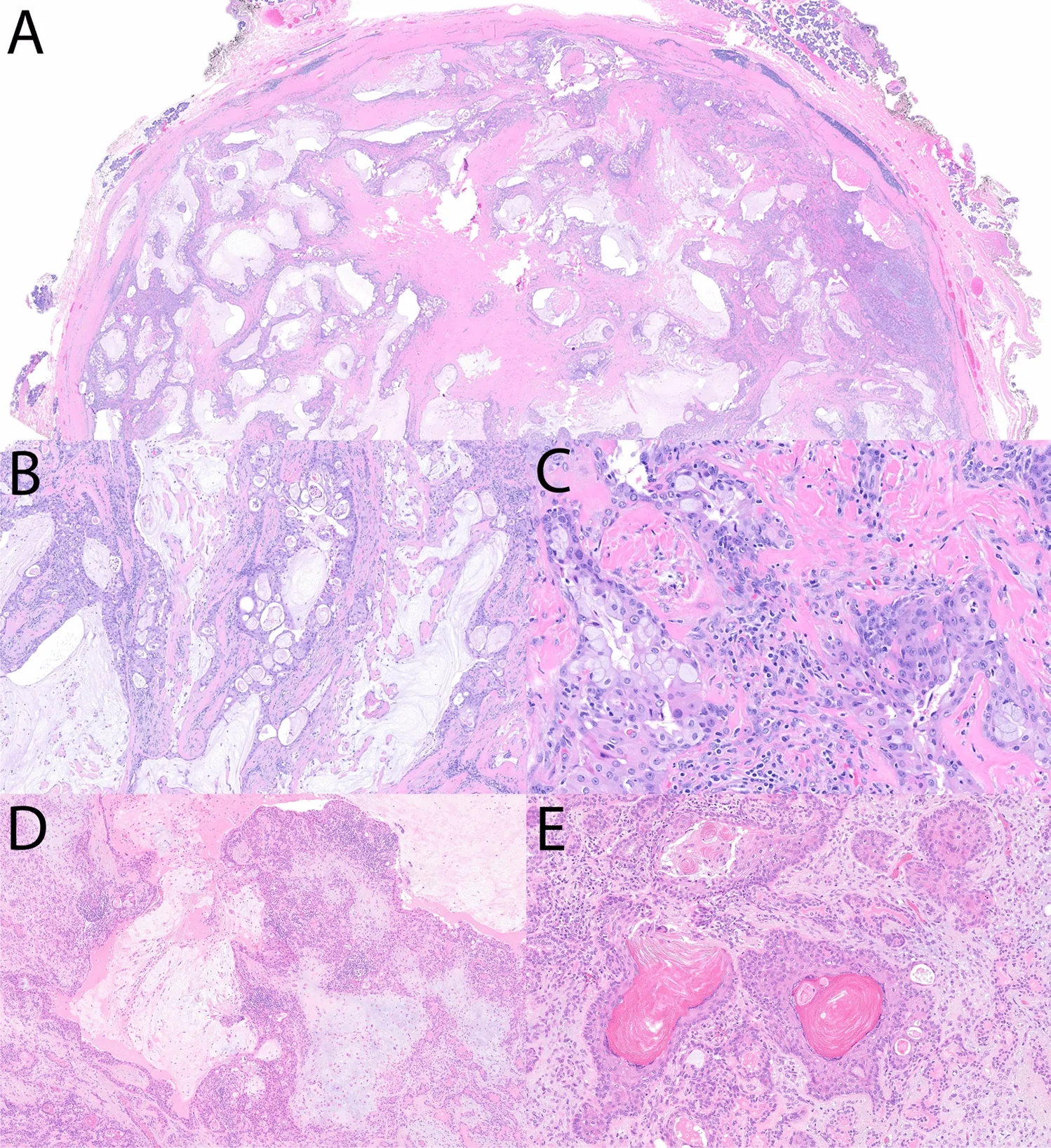
MEC-like areas were also well-circumscribed (**A**) and consisted of cystic squamous nests with abundant extracellular and intracellular mucin (**B**-**C**). MEC-like areas intermingled with conventional PA areas (**D**). Overt keratinizing squamous differentiation was focally identified (**E**)

Histologically, all tumors were well-circumscribed and clearly demarcated from the background parotid parenchyma (Fig. 1a-b). All tumors demonstrated characteristic histologic features of PA, namely, a biphasic proliferation of cytologically benign ductal and myoepithelial cells of variable cellularity, within chondromyxoid or fibrous stroma. Additionally, all tumors demonstrated areas of cystic squamous differentiation, mucin pools, and mucous cells closely mimicking MEC, and these areas comprised 25–80% of the tumors (Fig. 2). MEC-like areas admixed with conventional PA areas and did not form distinct “clonal” nodules. Additionally, keratinizing squamous differentiation was identified in all cases. No cytologic atypia, elevated mitotic activity, necrosis, or infiltrative-type growth was identified in any MEC-like areas.

**Fig. 3:**
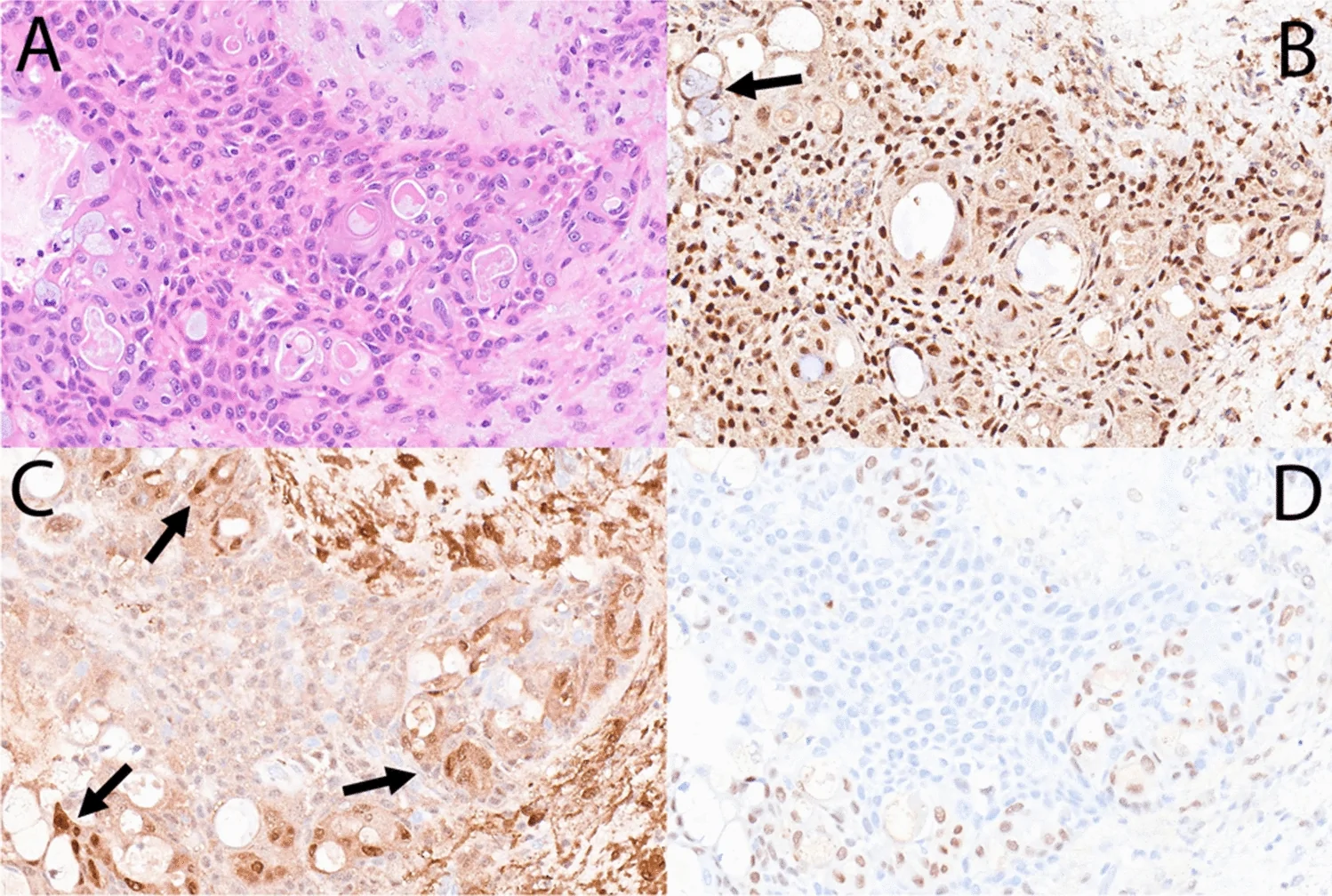
MEC-like areas (**A**) demonstrated immunohistochemical findings in support of a myoepithelial phenotype in the form of diffuse positivity for *PLAG1* (**B**, including in mucous cells (arrow)) as well as patchy positivity for S100 (**C**, arrows) and SOX10 (**D**) in tumor cells

The immunohistochemical and histochemical workup varied for each tumor and is detailed in Table 1. Among immunohistochemical and histochemical stains performed in more than one tumor, tumors were positive for CK7 (2/2), p63 (2/2, in myoepithelial cells and MEC-like areas), p40 (3/3, in myoepithelial cells and MEC-like areas), S100 (3/3, in myoepithelial cells and focally in MEC-like areas), SOX10 (2/2, in myoepithelial cells and focally in MEC-like areas),* PLAG1* (4/4, including in MEC-like areas), and mucin (3/3) (Fig. 1c, Fig. 3). Ki-67 was performed in 2 cases and was less than 5% in each.

Each tumor was subjected to next-generation sequencing in the course of routine clinical care (Table 2). All tumors harbored at *MALAT1::PLAG1* fusion (number of supporting reads ranged from 29–2043). The fusion involved *MALAT1* exon 1 or exon 2 paired with *PLAG1* exon 2 or exon 3. In the case evaluated by whole transcriptome sequencing (case 5), tumor mutational burden (TMB) was low (7 mutations/Mb) and the tumor was microsatellite stable (MSS). No *MAML2* or *EWSR1* rearrangements, other canonical MEC-driver alterations, any other fusion transcripts, or any oncogenic variants were identified in any cases.

**Table 2:** Molecular findings in *MALAT1::PLAG1*-rearranged pleomorphic adenoma

	Detected fusions	Involved exons/introns	*MAML2* or *EWSR1* alteration
1	*MALAT1::PLAG1*	*MALAT1* exon 2 (NR_144568.1)/ *PLAG1* exon 2 (NM_002655.3)	Not identified
2	*MALAT1::PLAG1*	*MALAT1* exon 2 (NR_144568.1)/ *PLAG1* exon 2 (NM_002655.3)	Not identified
3	*MALAT1::PLAG1*	*MALAT1* exon 1 (NR_002819.4)/ *PLAG1* exon 2 (NM_002655.3)	Not identified
4	*MALAT1::PLAG1*	*MALAT1* exon 2 (NR_144568.1)/ *PLAG1* exon 3 (NM_002655.3)	Not identified
5	*MALAT1::PLAG1*	*MALAT1* exon 2 (NR_144568.1)/ *PLAG1* exon 2 (NM_002655.3)	Not identified

## Discussion

In this series of 5 PA with cystic squamous and mucinous differentiation, benign cytomorphology, circumscription/encapsulation,* MALAT1::PLAG1* fusion, and absence of *MAML2* (or *EWSR1*) alteration, we affirm that co-occurrence of squamous and mucinous elements is within the histologic spectrum of PA. These 5 cases, in addition to the 2 recently reported cases in the literature, suggest a novel recurrent phenotype associated with *MALAT1::PLAG1* fusion [18, 19]. In 2 patients with follow-up from the present cohort and a third with follow-up reported by Wang, et al [19], there has been no evidence of recurrence or adverse events in 3 patients over follow-up periods of 8–25 months. Though these 7 cases point to an emerging genotype–phenotype correlation, more work is needed to determine the full histologic spectrum of *MALAT1::PLAG1*-rearranged PA as well as to determine if PA with squamous and/or mucinous metaplasia harbors additional fusion transcripts. To date, genotype–phenotype correlations in PA remain underexplored and are limited to *HMGA2-*rearranged PA with canalicular morphology, myoepithelial-predominant growth, CK7+/S100+/SOX10+/p63+/p40-/HMGA2+ immunophenotype, and frequent *HMGA2::WIF1* fusion transcripts, though alternate fusion partners with this phenotype and alternate morphologies with this genotype have been described [23–25].

The resemblance of areas in *MALAT1::PLAG1*-rearranged PA to MEC may not be coincidental. *MALAT1 *(Metastasis Associated Lung Adenocarcinoma Transcript 1) is a long non-coding RNA with established roles in splicing regulation and epithelial plasticity, and recent studies have suggested roles in cancer development, progression, and response to therapy [26]. *MALAT1 *overexpression has been reported across a variety of human malignancies [27]. Its fusion with *PLAG1* may dysregulate transcriptional programs governing squamous and mucinous differentiation, which may account for the distinctive phenotype observed. Although functional validation is beyond the scope of the present study, this hypothesis warrants further investigation with transcriptomic profiling, organoid models and methylation-based classifier studies to assess whether the *MALAT1* promoter specifically drives squamous and mucinous differentiation.

The clinical implications of accurate diagnosis in salivary gland neoplasms are significant. Misclassification of *MALAT1::PLAG1*-rearranged PA as MEC may result in unnecessary interventions, including selective neck dissection or adjuvant radiation and/or chemotherapy. The diagnostic challenge is most significant on small biopsy specimens or FNA biopsies, where conventional PA areas may be underrepresented. In one case in the current series (case 5), the initial biopsy consisted of mucin pools apparently dissecting between fibrous stroma, raising concern for an infiltrative mucinous process. Following correlation with the histologic findings of the resected tumor and molecular results, the findings were consistent with PA with squamous and mucinous metaplasia, harboring a *MALAT1::PLAG1* fusion transcript. Of note,* MALAT1* maps to chromosome 11q13 [28], which is relatively close to* MAML2* at 11q21 [29]; this chromosomal proximity may technically create interpretive challenges in FISH-based assays, highlighting the value of RNA sequencing for definitive fusion characterization when pursing molecular testing in this setting.

An important diagnostic consideration in the routine evaluation of PA is carcinoma ex pleomorphic adenoma (CXPA), defined as a malignant epithelial neoplasm arising from a pre-existing PA, especially since the presence of a PA-associated gene fusion would not discriminate between benign and malignant in this setting [1, 2]. The current WHO classification recommends subtyping the malignant component of CXPA according to a recognized salivary gland carcinoma, most commonly salivary duct carcinoma or myoepithelial carcinoma, with documentation of the extent of invasion [30]. It must be acknowledged that, in addition to some morphologic resemblance of *MALAT1::PLAG1*-rearranged PA to MEC, the absence of* MAML2* rearrangement alone would not categorically exclude MEC in all contexts. In the setting of CXPA, precedent exists for absence of a tumor-specific molecular event, namely, in the setting of epithelial-myoepithelial carcinoma ex PA which is known to occur without its characteristic HRAS mutation [31, 32]. However, numerous features still argue against consideration of MEC ex PA. Conceptually, the absence of capsular invasion, cytologic atypia, mitotic activity, or tumor necrosis would restrict any diagnostic consideration to “intracapsular low-grade MEC ex PA,” at most. Moreover, MEC-like foci histologically intermingle with conventional PA foci, instead of forming distinct clonal nodules as has been described, for example, in myoepithelial carcinoma ex PA [33]. And keratinizing squamous differentiation, which is considered an exceptionally rare phenomenon in MEC, can be readily seen in *MALAT1::PLAG1*-rearranged PA [34]. Immunohistochemically, focal S100 and SOX10 immunopositivity was observed in MEC-like areas in all cases in which these stains were performed, but S100 immunopositivity is considered rare in MEC and SOX10 immunopositivity has been reported only in a subgroup of *MAML2*-rearranged MEC with distinctive histologic features [35, 36]. Additionally, previous work has demonstrated the utility of S100 and SOX10 immunohistochemistry in demonstrating a myoepithelial phenotype in PA with mucocytes [4]. Altogether, the histologic, immunohistochemical, and molecular features argue against interpretation as MEC ex PA. And in clinical practice, wide availability of immunohistochemistry for S100 and SOX10 represents a practical and cost-effective first-line approach to confirming a diagnosis of PA with squamous and mucinous metaplasia, particularly in small biopsy specimens when diagnoses of MEC or metastatic squamous cell carcinoma may be considered.

The majority of PAs harbor rearrangements in* PLAG1* (8q12) or* HMGA2* (12q14.3), rendering these transcription factors under the control of heterologous promoters [1, 2]. Frequently reported partner genes for *PLAG1* include* CTNNB1*, *FGFR1*, *LIFR*,* CHCHD7*, *TCEA1*, *NFIB*, and *BOC* [37–41]. From a molecular perspective, the oncogenic mechanisms underlying *PLAG1*-rearranged PA and *MAML2*-rearranged MEC are distinct. The human *PLAG1* gene, located on chromosome 8q12, consists of 5 exons; the coding sequence is confined to exons 4–5, while exons 1–3 constitute the 5' untranslated region [42]. Recurrent *PLAG1* fusions in PA result from promoter substitution, in which the 5' non-coding exons are replaced by sequences from a heterologous partner gene, leaving the coding sequence intact [43].* PLAG1* rearrangements dysregulate *IGF2* and related growth factor signaling pathways. On the other hand, the* CRTC1::MAML2* fusion alters Notch signaling as well as the p16-CDK4/6-RB pathway [29, 44]. No known mechanistic study has described signaling pathway convergence with what is observed in PA, undermining the biological plausibility of MEC arising as the malignant component of CXPA in the absence of an identifiable molecular event.

Genotype–phenotype correlations are rarely “air-tight” and, of note, during manuscript revision, a PA with squamous and mucinous differentiation was encountered by one of the authors (DAK) that was found to harbor a novel *RNF170::FGFR1* fusion, suggesting that, indeed, *MALAT1::PLAG1* fusion may not universally account for this phenotype. Additionally, another PA occurring on the hard palate was found during manuscript revision by one of the authors (IL) to harbor *MALAT1::PLAG1* fusion but histologically demonstrated small nests of squamous differentiation, only, and more closely represented sialadenopapillary ductal tumor (sialadenopapilliferum-like intraductal papillary tumor) than MEC by histology. Reports of additional cases will be necessary to refine the histologic and molecular spectrum of the cases reported in this series.

In conclusion, PA can rarely demonstrate cystic squamous and mucinous differentiation, which may correlate with the presence of *MALAT1::PLAG1*-rearrangment. Although some morphologic overlap with MEC may exist, the balance of histologic, immunohistochemical, and molecular findings can help to support the diagnosis of PA (without a carcinomatous component), and awareness of this diagnostic pitfall is necessary to avoid overdiagnosis in FNA or core needle biopsies.

## Data Availability

No datasets were generated or analysed during the current study.

## Code Availability

No software application or custom code has been used in the preparation of this manuscript.
